# Effect of cation and anion sizes of additive ionic liquid on the crystal structure of poly(vinylidene fluoride) nanofiber†

**DOI:** 10.1039/d3ra01917a

**Published:** 2023-04-17

**Authors:** Hanako Asai, Hiroyuki Saga, Ryuto Saito, Koji Nakane

**Affiliations:** a Frontier Fiber Technology and Science Course, Graduate School of Engineering, University of Fukui 3-9-1 Bunkyo Fukui 910-8507 Japan h_asai@u-fukui.ac.jp

## Abstract

We investigated the additive ionic liquid (IL) type dependence on the crystal structure of poly(vinylidene fluoride) (PVDF) nanofibers. As additive ILs, we used imidazolium based ILs with different cation and anion sizes. From differential scanning calorimetry (DSC) measurements, we found that there is a proper amount for the additive IL to promote PVDF crystallization, and the proper amount is influenced by the cation size, not by the anion size. In addition, it was found that IL itself inhibited the crystallization, but IL can promote crystallization under the presence of DMF.

## Abstract 2

We investigated the additive ionic liquid (IL) type dependence on the crystal structure of poly(vinylidene fluoride) (PVDF) nanofibers.

## Graphical abstract

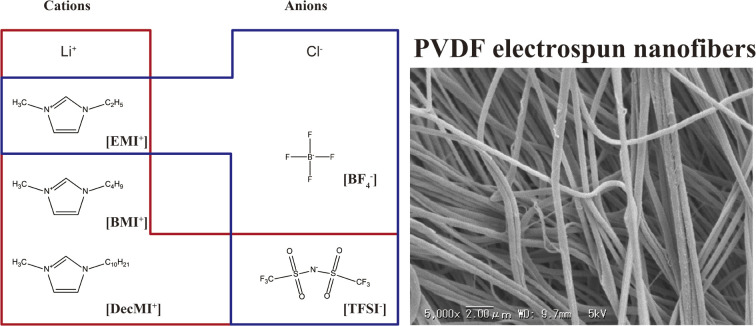

## Introduction

Poly(vinylidene fluoride) (PVDF) is a well-known piezoelectric polymer. However, the distinct piezoelectricity of PVDF appeared only when the electroactive crystal phase was dominant in the PVDF sample. There are three main crystal phases for PVDF: first, the α-phase exhibits a *trans*–*gauche*–*trans*–*gauche*′ (TGTG′) conformation of macromolecular chains.^1^ It is nonpolar and does not exhibit ferroelectric behavior. The β-phase exhibits an all-*trans* (TT) conformation.^1^ The γ-phase can be considered an intermediate between the α- and β-phases, and it exhibits a TTTGTTTG′ conformation.^1^ Because the α-phase is the most thermodynamically stable structure, subjecting the α-phase PVDF to mechanical or electrical treatments is necessary to obtain β-phase-dominant PVDF.^2–4^ In this study, we call β- and γ-phases “electroactive phase”.

On the other hand, electrospinning (ES) is a known as the processing method, where PVDF is transformed to β-phase dominant state simultaneously with the nanofiber mat form.^5,6^ In the ES process, the PVDF solution is ejected from a spinning nozzle and drawn to a metal collector by applying a high voltage. The solvent in the PVDF solution evaporated during the spinning process, resulting in the formation of a nanofiber mat. The obtained piezoelectric PVDF nanofiber mats were used as flexible nanogenerators.^5–7^

In our previous paper, we investigated the effect of the addition of ionic liquid (IL) 1-ethyl-3-methyl-imidazoliun bis(trifluoromethanesulfonyl) imide ([EMI^+^][TFSI^−^]), on the crystal structure of PVDF nanofibers prepared by ES.^8^ From this previous study, we found that the addition of [EMI^+^][TFSI^−^] promoted electroactive phase formation in PVDF nanofibers. Some researchers have also reported the effects of IL addition on PVDF systems.^7,9–11^ For example, Pickford *et al.* investigated the effects of an additive IL (1-allyl-3-methylimidazolium chloride (AMIM)) and processing methods on the crystalline structure of PVDF.^9^ In terms of salt addition, Sobola *et al.* reported that calcium and magnesium nitrates enhanced the-phase of PVDF nanofibers.^12^ However, the influence of the cation and anion type of the additive ILs was unclear. Barbosa *et al.* studied electrospun PVDF fiber membranes doped with different ILs that shared the same anion and their potential as separator membranes for battery applications.^11^ Although various types of cations were used, the effect of the ion size was not discussed well. Considering that the electroactive phase formation effect by the additive occurs due to intermolecular interactions such as hydrogen bonding between the additive and the PVDF chain,^13^ the size difference between the cation and anion of the additive IL should influence the intermolecular interaction with the PVDF chain.

In this study, we investigated the effect of the cation and anion types of the additive ILs on the formation of the electroactive phase of PVDF nanofibers. For the cation-varied series, we selected ILs consisting of imidazolium-based cations with different alkyl chain lengths and TFSI^−^ anions. For the anion-varied series, ILs consisting of the EMI^+^ cation and different-sized anions were investigated.

## Experimental

### Materials

**Fig. 1:**
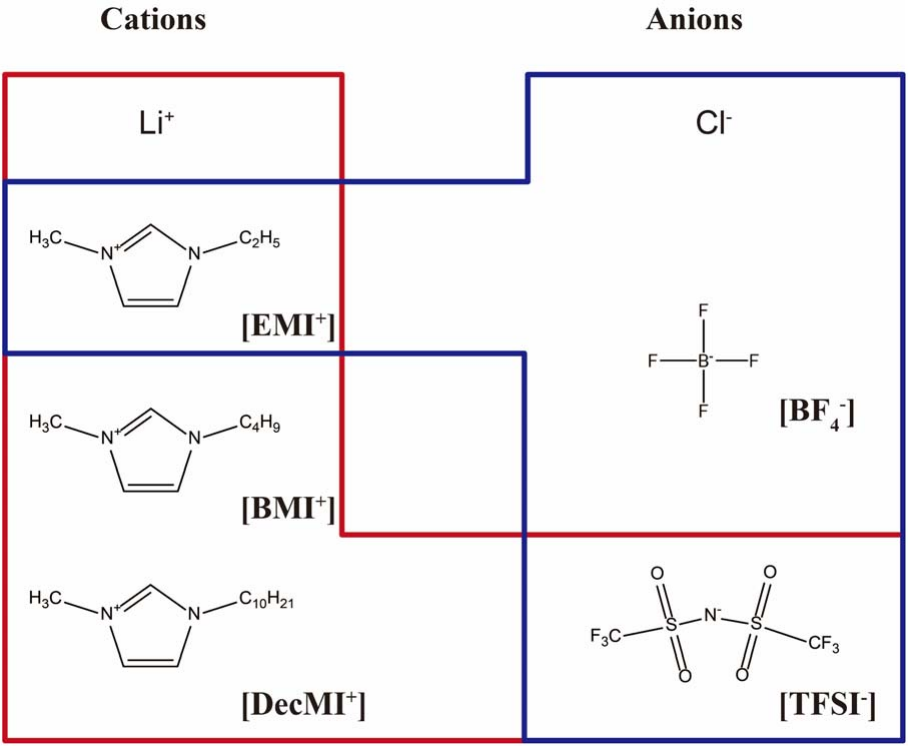
ILs used in this study. The experimental results of the [EMI^+^][TFSI^−^] is referred from the ref. 8. The combinations surrounded by the red line are “cation-varied series”, and those surrounded by the blue line are “anion-varied series”.

PVDF pellets (*M*_w_ = 275 000 g mol^−1^) were procured from Sigma-Aldrich (Tokyo, Japan). Based on a previous study,^8^ in which water also affected the crystal structure of PVDF, super dehydrated DMF (Fujifilm Wako Pure Chemical Corporation, Osaka, Japan) was utilized as the solvent for the spinning solution of PVDF. The ILs used in this study are summarized in Fig. 1. All the ILs were purchased from Tokyo Chemical Industries.

### Preparation of the nanofiber mat samples

PVDF was dissolved in super-dehydrated DMF, and then each IL was successively added to the solution. The PVDF concentration was fixed at 26 wt%, and the amount of ILs was varied against the total weight of the mixed solvent. In this paper, the IL concentration is indicated in mol% to show the actual number of IL molecules. In Table S1,† the correspondence between mol% and wt% are summarized. The mixtures were magnetically stirred at 70 °C for a minimum of 2 h until homogeneous solutions were obtained. Each resulting solution was transferred to a syringe and loaded into the ES apparatus. The spinning conditions were as follows: spinning volume of 2 mL; nozzle-to-collector distance of 20 cm; injection rate of 0.5 mL h^−1^. The applied voltage was set to 20 kV. After spinning, the nanofibers were peeled off from the collector and maintained under vacuum. All experiments were performed at room temperature.

### Characterization

Differential scanning calorimetry (DSC) curves were obtained in air using a calorimeter (DSC-60; Shimadzu Corporation, Kyoto, Japan) from 30 to 250 °C at 10 °C min^−1^.

Scanning electron microscopy (SEM) was performed using a Keyence scanning electron microscope (VE-9800; Keyence Co., Ltd, Osaka, Japan) at 5 kV. The samples were sputter-coated with gold using an ion coater (SC-701; Sanyu Electron Co. Ltd, Tokyo, Japan). The average and standard deviations (*D* and *σ*, respectively) of the fiber diameters were determined from 100 measurements using Photo Ruler software.

Attenuated total reflectance Fourier-transform infrared (ATR-FTIR) spectroscopy data were obtained at room temperature using an IR spectrometer (IR Affinity-1; Shimadzu Corporation) equipped with a single reflection ATR accessory (MIRacle 10; Shimadzu Corporation) containing a diamond/ZnSe crystal. The measurements were performed from 700 to 4000 cm^−1^.

## Results and discussion

### SEM observation

**Fig. 2:**
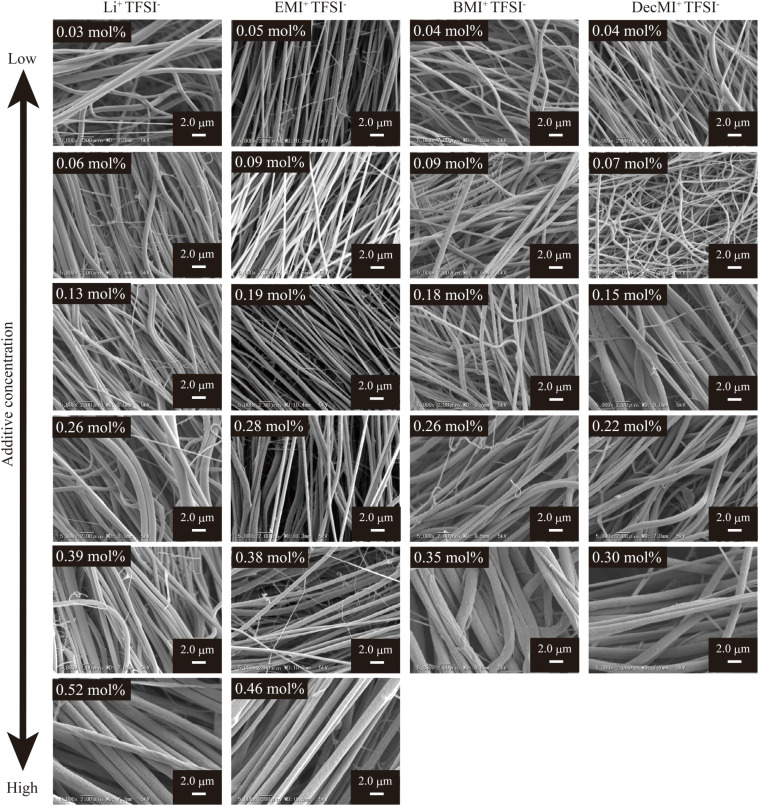
SEM images of the cation-varied series nanofiber mats spun from various additive IL concentrations. The PVDF concentration was fixed at 26 wt%.

**Fig. 3:**
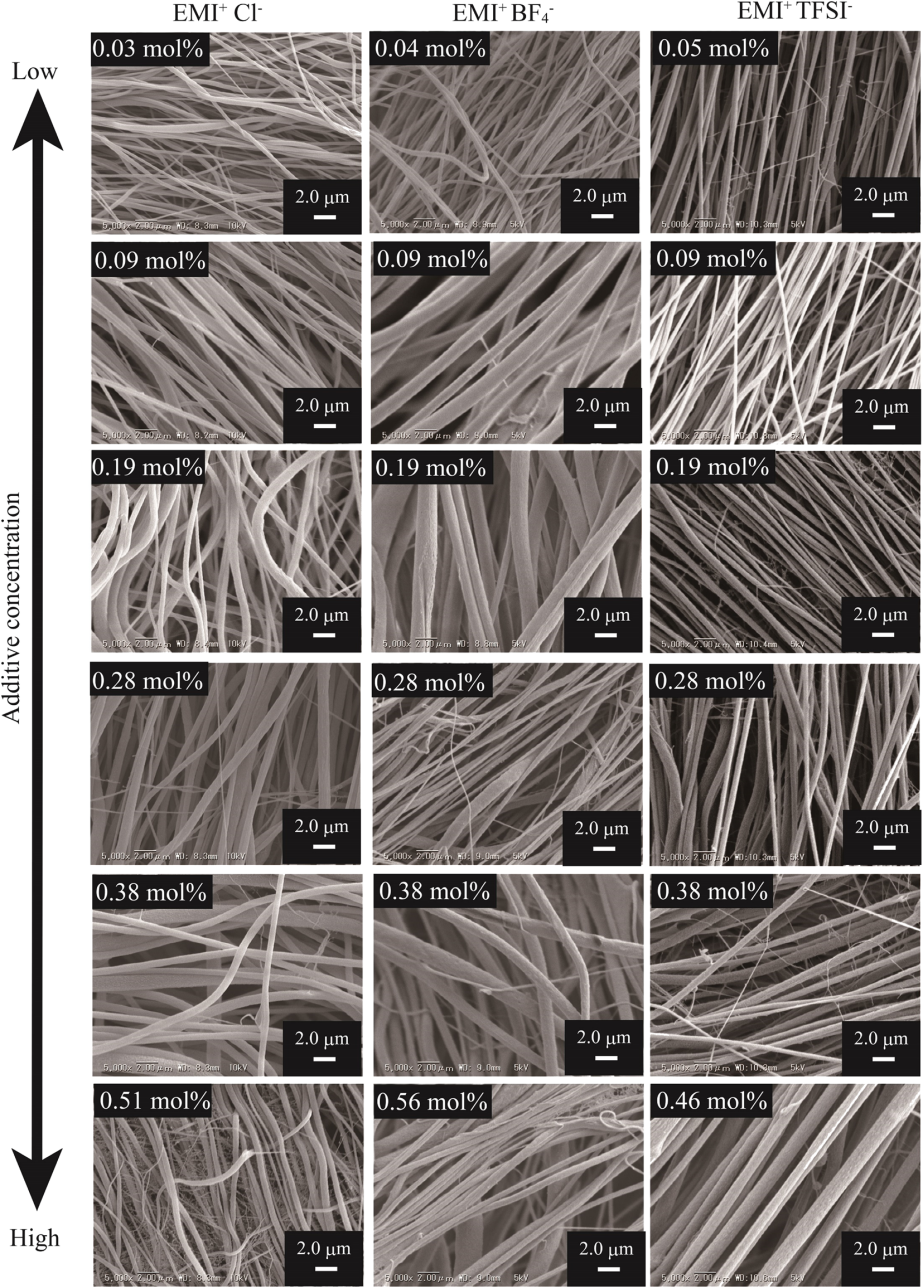
SEM images of the anion-varied series nanofiber mats spun from various additive IL concentrations. The PVDF concentration was fixed at 26 wt%.

**Fig. 4:**
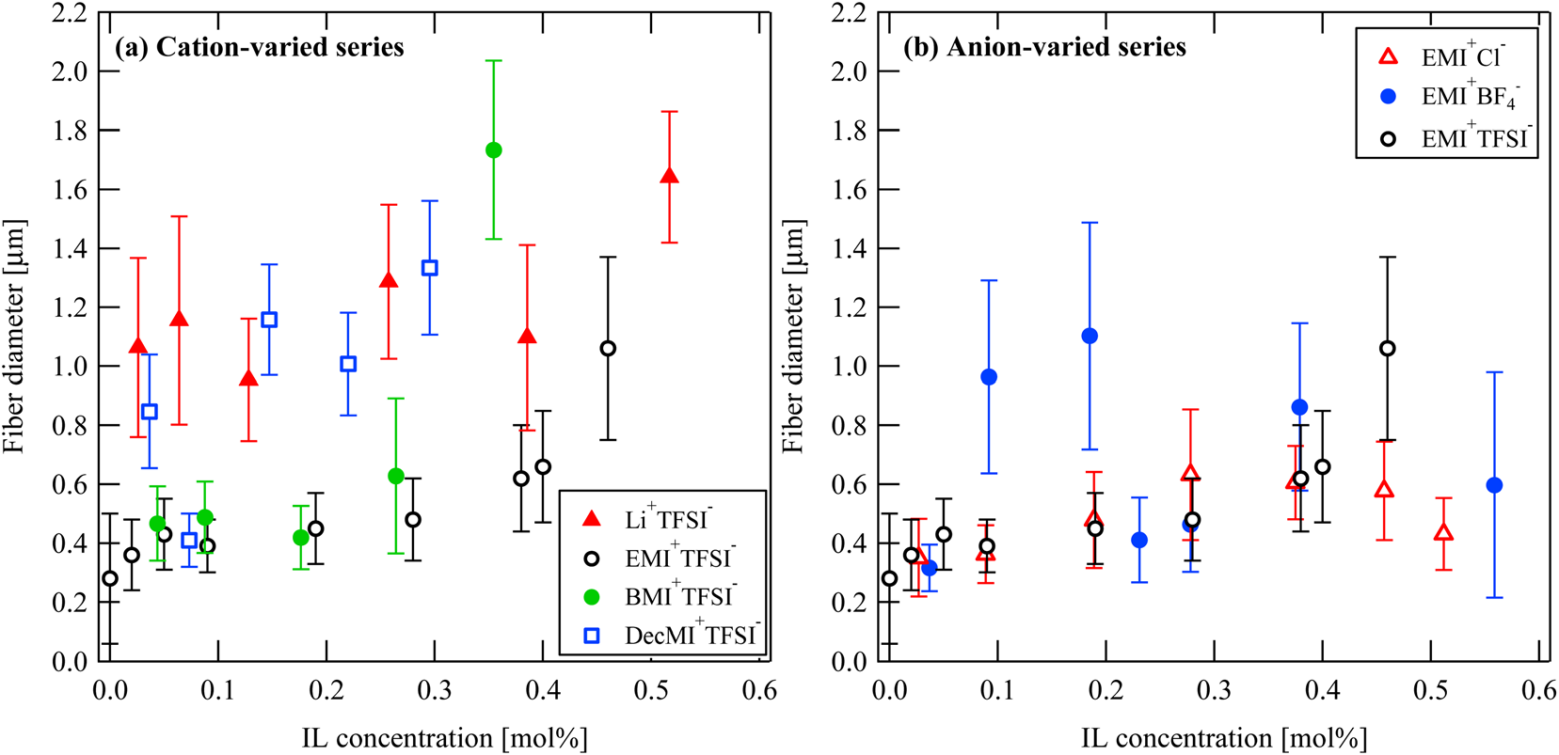
IL concentration and type dependences on the nanofiber diameter: (a) cation-varied series and (b) anion-varied series.

Fig. 2 and 3 show the SEM images of the nanofibers obtained from the spinning solutions containing different concentrations of cation-varied and anion-varied IL additives, respectively. We evaluated the fiber diameters of each series. Fig. 4(a) shows the variation in fiber diameters for the cation-varied series. The fiber diameter of the cation-varied series increased with IL concentration. The Li^+^TFSI^−^ and DecMI^+^TFSI^−^ series showed relatively larger fiber diameters. For the anion-varied series (Fig. 4(b)), the difference in fiber diameter between the IL types was not large compared to the cation-varied series. It is known that the addition of salt increases the thickness of the ES nanofibers because the viscosity of the spinning solution increases with the addition of salt.^14,15^ However, for both series, very thin fibers appeared between the observed fibers. These thin fibers were formed because of the charge repulsion introduced by the addition of the IL.^7^ Therefore, the fiber diameter was affected by these two opposing effects.

### DSC measurement

**Fig. 5:**
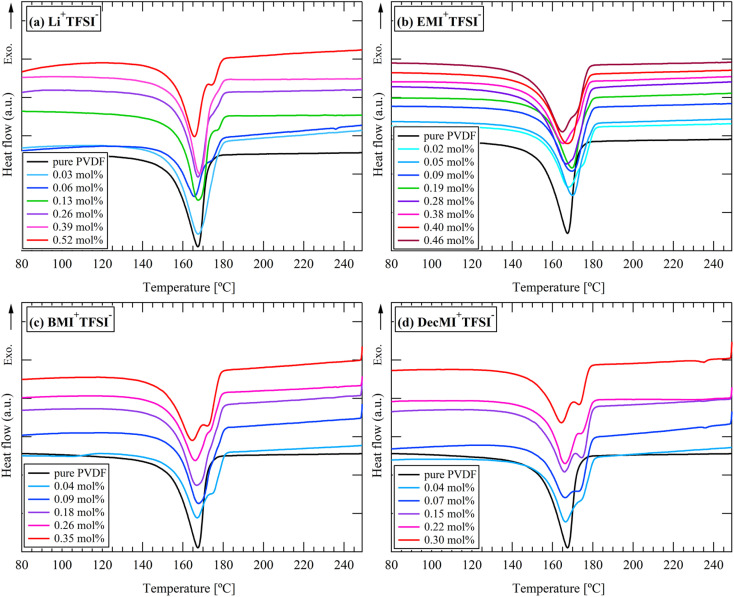
DSC curves for cation-varied series during heating process: (a) Li^+^TFSI^−^, (b) EMI^+^TFSI^−^, (c) BMI^+^TFSI^−^, and (d) DecMI^+^TFSI^−^.

**Fig. 6:**
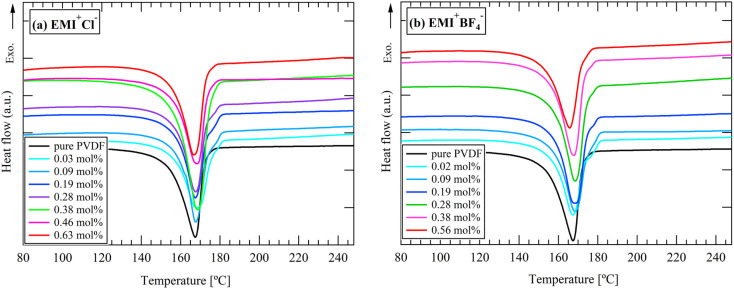
DSC curves for anion-varied series during heating process: (a) EMI^+^Cl^−^, (b) EMI^+^BF_4_^−^.

Fig. 5 and 6 show the DSC curves of the cation- and anion-varied series, respectively. An endothermic peak was observed at approximately 165–180 °C in all results, indicating the fusion of the crystal. For the cation-varied series, two peaks were observed at 165–168 °C and 173–177 °C. For the anion-varied series, one peak was observed at approximately 165–168 °C and a small shoulder was observed at approximately 175 °C. These results indicate that the effect of cation size on the higher melting peak appears to be larger than that of anion size. Considering that the higher melting temperature can be attributed to β- or γ-crystals,^16,17^ the cation-varied series consisting of TFSI^−^ may contain a higher ratio of electroactive phases. However, as shown in the FT-IR results, the electroactive phase contents of the two series were not significantly different. Considering that in PVDF and in most semicrystalline polymers, the melting temperature has been linked to the lamellar thickness,^16^ the cation-varied series may have thicker lamellae.

The crystallinity (*χ*_c_) was evaluated from *χ*_c_ [%] = Δ*H*/ΔH* × 100. Δ*H* and ΔH* are the heats of fusion obtained from DSC measurements and the perfect crystal, respectively. Additionally, 104.7 J g^−1^ was employed as the ΔH* value.^18^

**Fig. 7:**
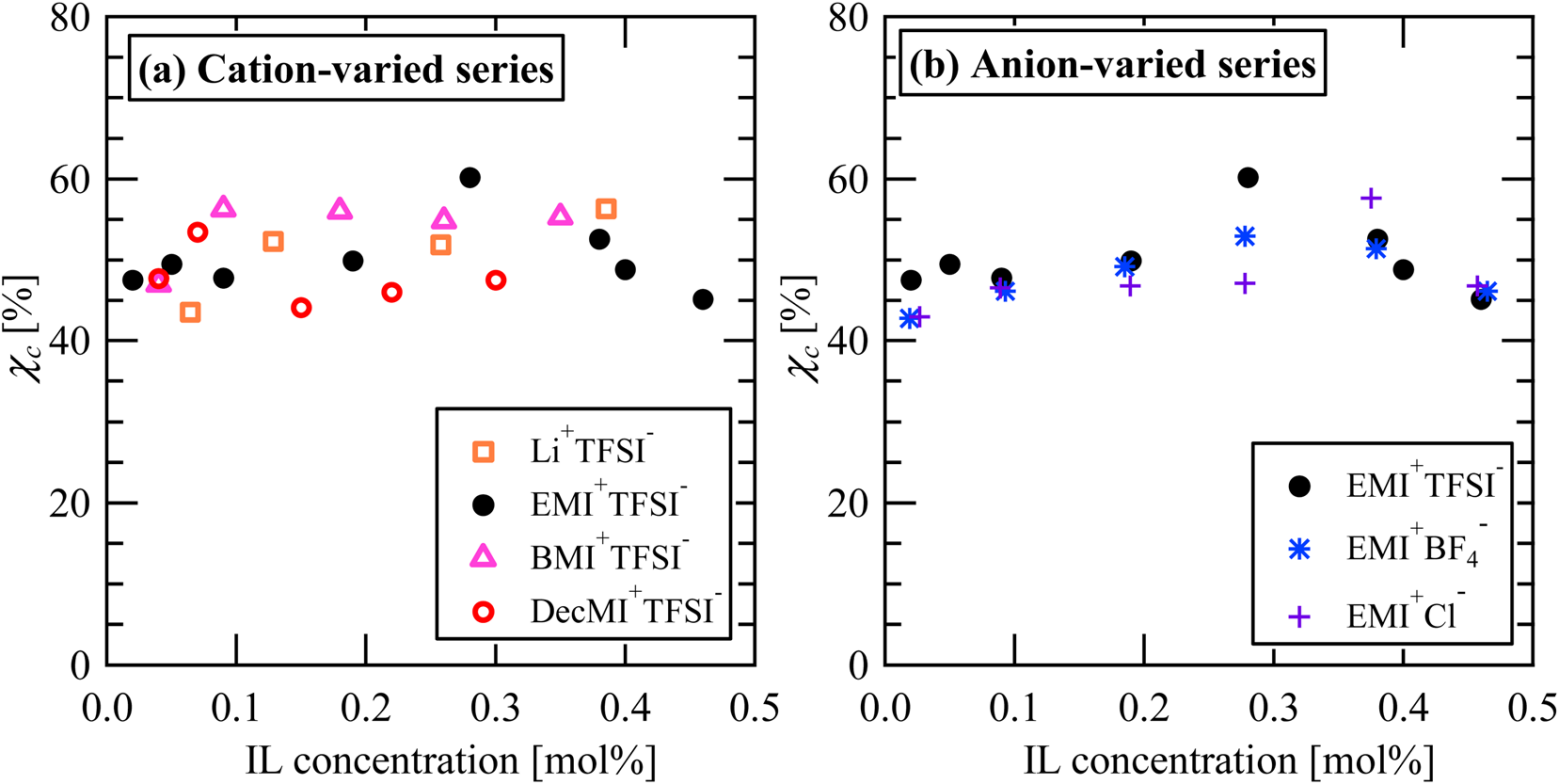
Additive IL concentration dependences on the crystallinity, *χ*_c_ for (a) cation-varied and (b) anion-varied series.

The evaluated *χ*_c_ values are shown in Fig. 7. As shown in Fig. 7, the cation type had a larger influence on the *χ*_c_ values than the anion type, indicating that the cation of the IL should have a larger effect on the crystal formation of PVDF nanofibers compared with the anion of the IL. Additionally, for the cation-varied series (Fig. 7(a)), the *χ*_c_ value showed a maximum with increasing IL concentration, and the maximum peak position shifted to the right as the cation size decreased. Therefore, it was found that there is an appropriate amount for each additive IL to promote crystallization, and the IL with a smaller cation size needs a larger amount. In contrast, Barbosa *et al.*^11^ reported that the *χ*_c_ value decreased with IL addition. This is because the amount of IL in their study was much larger than that in our case. In Fig. S1 and S2,† results of X-ray diffraction (XRD) measurements are also shown. Please note that the sample amounts were different between the data, because the nanofiber mats contained a lot of inter-fiber spaces. As shown in these figures, by the IL addition, the peak at 19.9° corresponding to the α-phase (110) reflections was shifted to 20.6° corresponding to the (110, 200) peak of the β-phase.^1^ These results were also consistent with the FT-IR results shown in later.

**Fig. 8:**
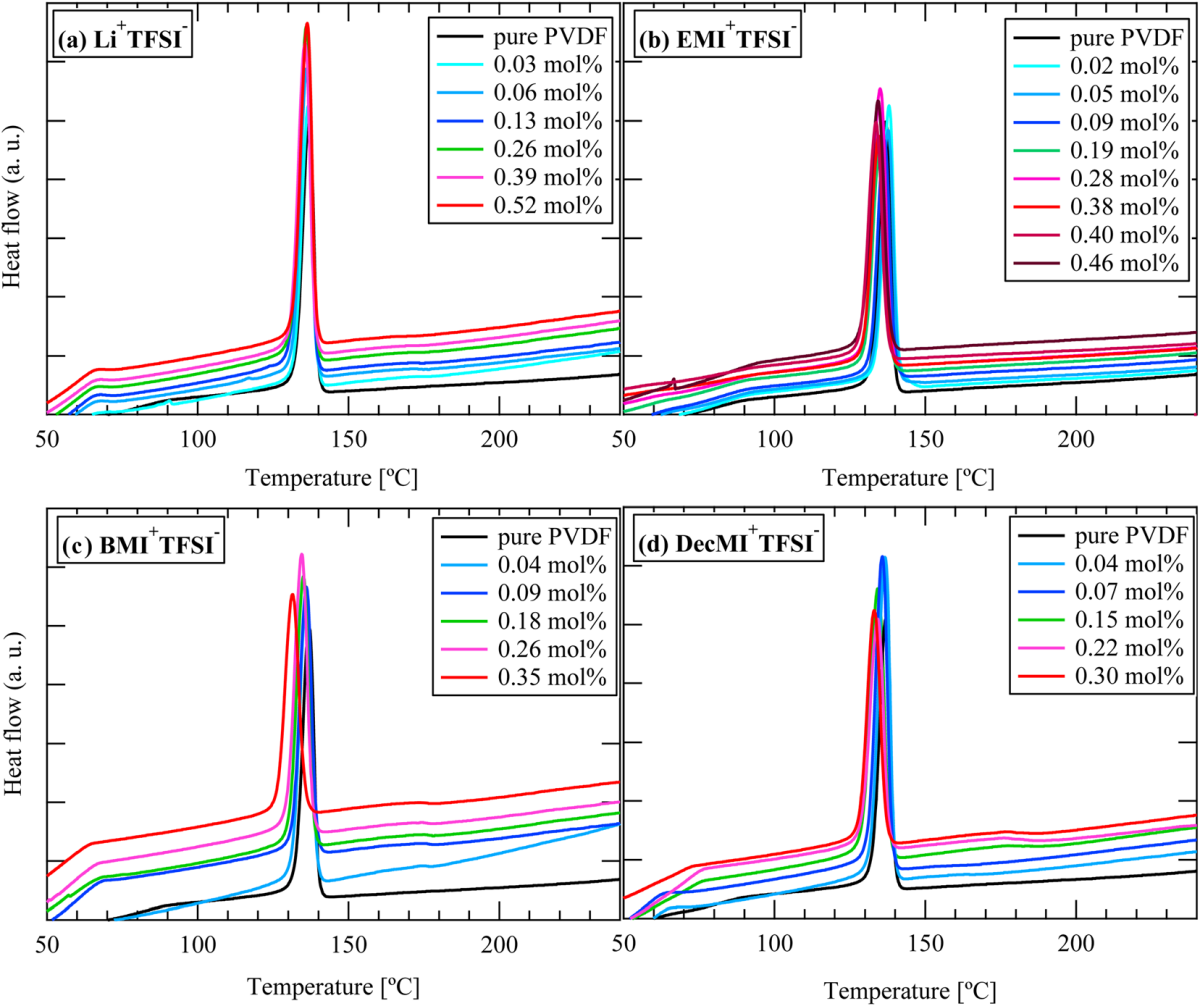
DSC curves for cation-varied series during cooling process: (a) Li^+^TFSI^−^, (b) EMI^+^TFSI^−^, (c) BMI^+^TFSI^−^, and (d) DecMI^+^TFSI^−^.

**Fig. 9:**
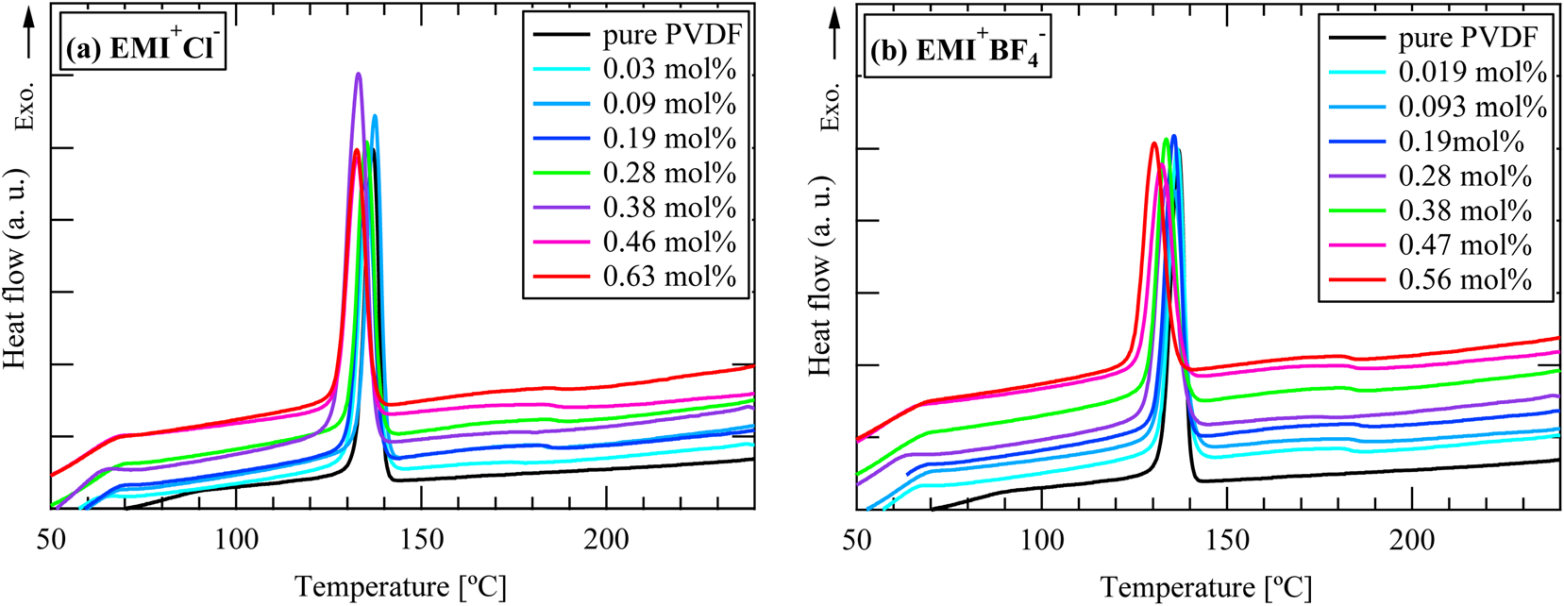
DSC curves for anion-varied series during cooling process: (a) EMI^+^Cl^−^ and (b) EMI^+^BF_4_^−^.

**Fig. 10:**
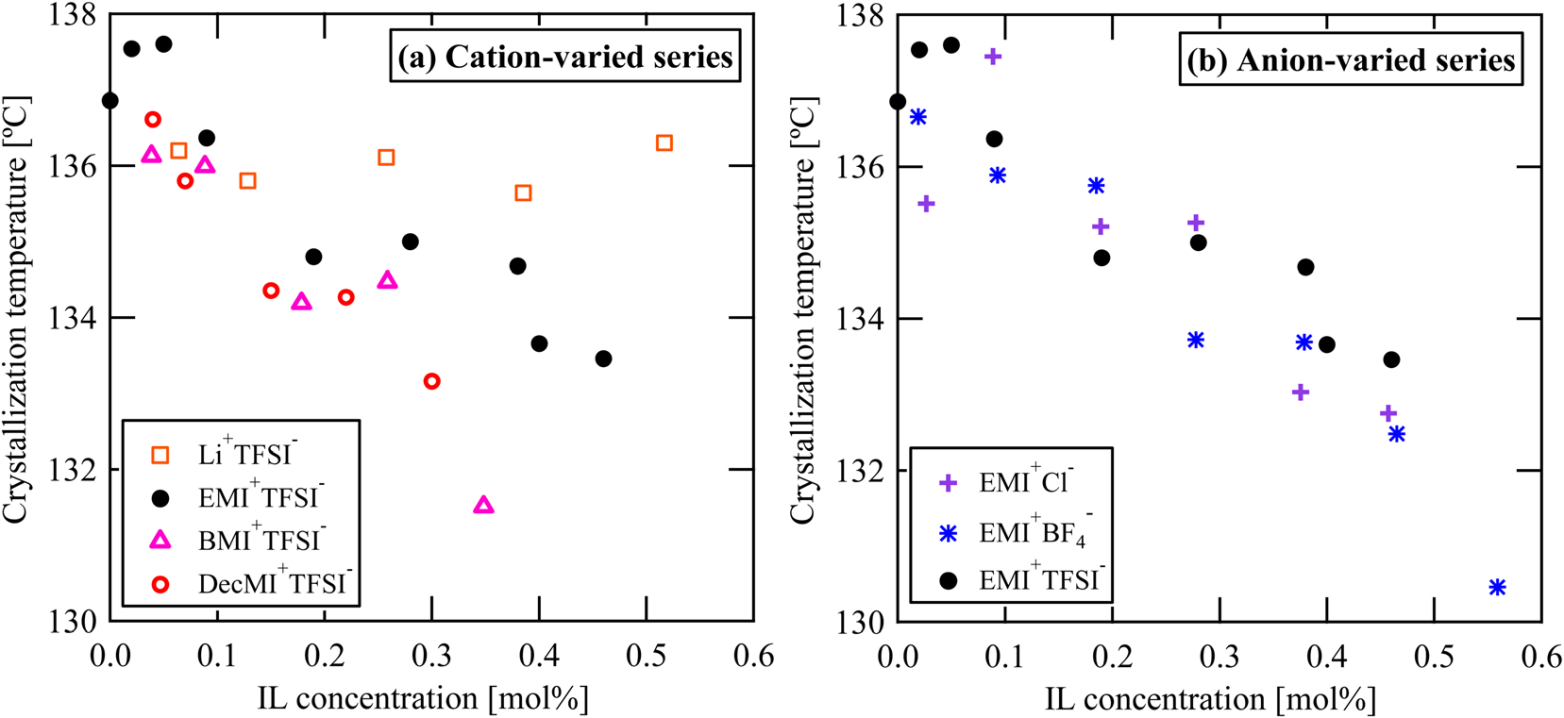
IL concentration and type dependences on the crystallization temperature: (a) cation-varied series, and (b) anion-varied series.

The DSC curves for the cation- and anion-varied series during the cooling process are summarized in Fig. 8 and 9, respectively. The samples did not have fibrous form during the cooling process because the melting temperature of PVDF was approximately 170 °C. Based on the position of the exothermic peak, the crystallization temperature was evaluated. All the observed exothermic peaks were single ones, differing from the heating process. Generally, α-phase crystal is formed during cooling process from melted state,^1^ the single exothermic peak can be attributed to α-phase crystal formation. Because ILs are nonvolatile, they should remain in the system even after the PVDF nanofibers melt upon heating. The obtained crystallization temperatures are shown in Fig. 10. For both the series, the crystallization temperature tended to decrease with increasing IL concentration. On the other hand, Indolia *et al.* reported that for a PVDF/ZnO nanocomposite system, the crystallization temperature increased with increasing ZnO content, which acted as a nucleating agent.^19^ Therefore, our results indicate that the ILs themselves do not promote the crystallization of PVDF as nucleation agents. Additionally, the influence of the cation size on the crystallization temperature was greater than that of the anion size. For the Li^+^TFSI^−^ system, the crystallization temperature was not affected by the Li^+^TFSI^−^ concentration, whereas for the DecMI^+^TFSI^−^ system, the crystallization temperature decreased significantly with increasing DecMI^+^TFSI^−^ concentration. However, this size effect was not clearly observed in the anion-varied series. According to a study by Silva *et al.*^20^, the EMI^+^ cation is preferentially coordinated to the –CF_2_ of the PVDF chain, while the anion interacts with the –CH_2_ of the PVDF chain. A cation with a longer alkyl chain inhibits the crystallization of PVDF, and its influence is larger than that of the anion.

From the data of Fig. 10, the IL inhibits the crystallization. However, the process shown in Fig. 10 did not contain DMF, while the ES nanofiber was prepared from a three-component system (PVDF, DMF, and IL). Therefore, the presence of DMF may be a key factor in the crystallization and phase formation of the nanofiber.

### FT-IR measurements

Fig. S3† shows the FT-IR spectra of the cation- and anion-varied series. The broken lines at 761, 840, 1234, and 1275 cm^−1^ in Fig. S3† correspond to the α-, β- and γ-, γ-, and β-phases, respectively.^21^

The relative fraction of the electroactive β- and γ-phases (*F*_EA_) of PVDF was evaluated from the FT-IR data using the following equation:^21^1
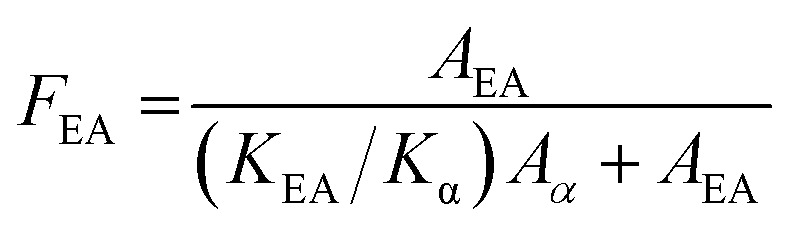
where *A*_α_ and *A*_EA_ are the absorbances at 761 and 840 cm^−1^, respectively, and *K*_α_ and *K*_EA_ are the absorption coefficients at the respective wavenumbers of 6.1 × 10^4^ and 7.7 × 10^4^ cm^2^ mol^−1^, respectively.^21^ According to Cai *et al.*,^21^*F*_EA_ can be further divided into the fractions of individual β and γ phases by using the FT-IR absorbance differences between the peak around 1275 cm^−1^ and the nearest valley at 1260 cm^−1^, and the peak at 1234 cm^−1^ and the nearest valley at 1225 cm^−1^, respectively. However, we could not evaluate *F*(β) and *F*(γ) values because the peak around 1234 cm^−1^ and the nearest valley around 1225 cm^−1^ were obscure for both series (Fig. S3†). Therefore, in this study, we only discuss *F*_EA_ values.

**Fig. 11:**
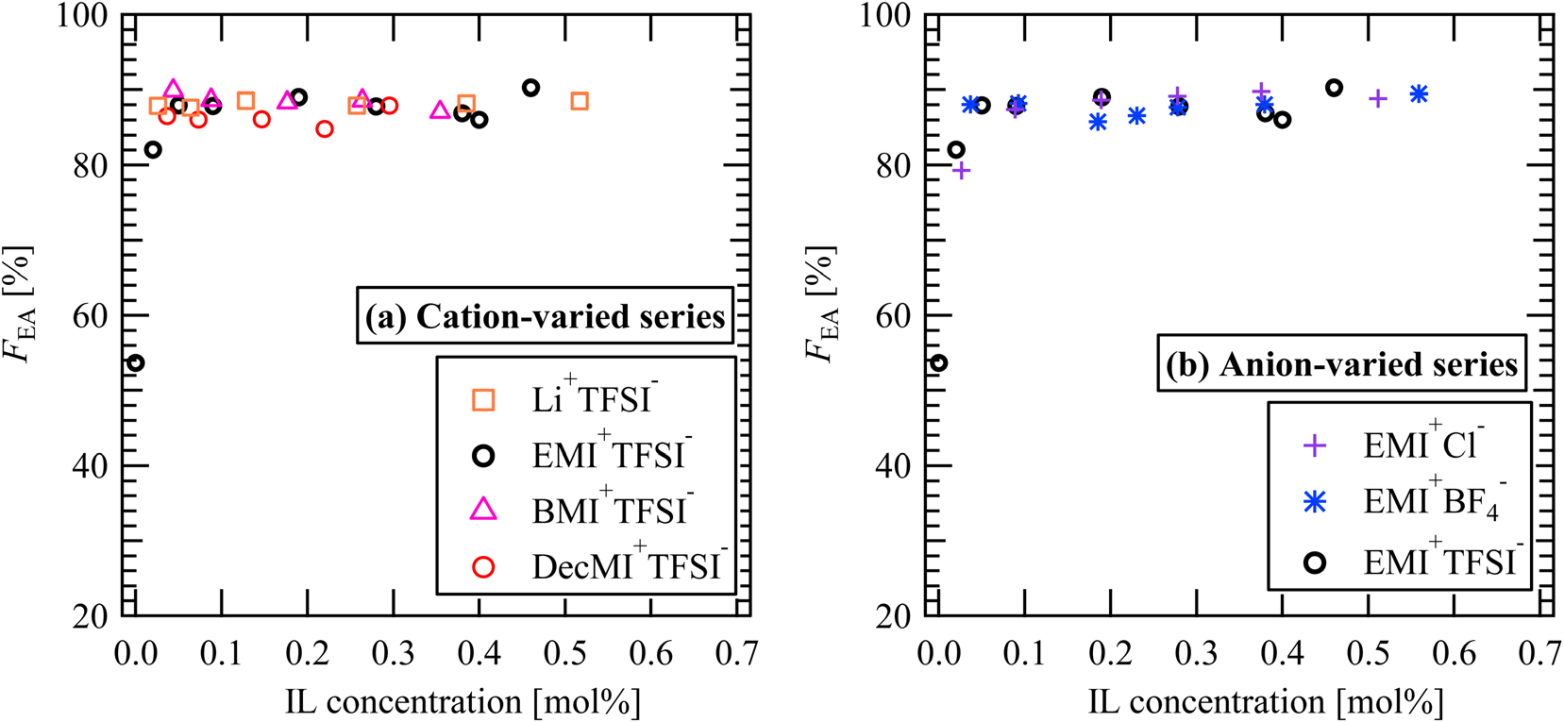
*F*_EA_ values for (a) cation-varied series and (b) anion-varied series.

Fig. 11 summarizes the *F*_EA_ values as a function of the amount of IL additive. Irrespective of the IL type, the *F*_EA_ values steeply increased with the addition of IL, but they did not change significantly with further IL addition. Considering the additive IL molar amount against PVDF, the IL interacts with PVDF, not one by one.

Fig. S4† shows the *F*_EA_ values of the film samples prepared using the bar-coat method. Compared with the nanofiber samples (Fig. 11), the effect of the IL addition on the electroactive phase formation was small. Based on this result, we conjectured that sample preparation time or high voltage during ES process is essential. To examine this hypothesis, we further compared the FT-IR spectra of the cast films, bar-coat films, and nanofibers (Fig. S6†). In Fig. S6,† the preparation time is longer in the order of cast films, bar-coat films, and nanofibers. For the nanofiber samples, a clear peak at 1275 cm^−1^ corresponding to the PVDF β-phase was observed; however, only broad shoulders were detected for the cast and bar-coat films. This result indicates that the β-phase tends to be formed during the ES process. Because the γ-phase is thermodynamically more stable than the β-phase, the crystalline structure may change from β- to γ- for the cast and bar-coat films, where preparation takes a long time and high voltage is not applied. Additionally, for the ES nanofibers, the 761 cm^−1^ peak corresponding α-phase disappeared by the addition of IL (Fig. S3 and S6†). Therefore, ES process with IL is proper method to improve β-phase fraction.

From these results, we conclude that the coexistence of DMF and IL is necessary to improve the crystallinity, but the IL itself does not promote crystallization. The effective additive amount of the IL depends on the cation size. To improve the β-phase, ES with IL is effective.

## Conclusions

We investigated the effect of the cation and anion sizes of the additive ionic liquids (ILs) on the crystal structure of PVDF nanofibers prepared by the electrospinning (ES) method. The important results of this work are described below:

(1) Under the presence of DMF, IL promote crystallization of PVDF, although IL itself inhibits the crystallization.

(2) There is a proper amount for the additive IL to promote PVDF crystallization. The proper amount is influenced by the cation size, not by the anion size.

(3) To improve the β-phase, ES with IL is effective.

## Author contributions

Hanako Asai made the experimental plans and wrote the manuscript. Hiroyuki Saga and Ryuto Saito carried out experiments and analysis of data. Koji Nakane advised the experiments and modified the manuscript. All authors have read and agreed to the published version of the manuscript.

## Conflicts of interest

There are no conflicts to declare.

## Supplementary Material

RA-013-D3RA01917A-s001
