# Supplementary material for: Effect of cation and anion sizes of additive ionic liquid on the crystal structure of poly(vinylidene fluoride) nanofiber
Source: RSC Adv. 2023 Apr 17;13(18):12000–8. doi: 10.1039/d3ra01917a (PMC10107745; doi:10.1039/d3ra01917a)
Supplement: RA-013-D3RA01917A-s001 [file RA-013-D3RA01917A-s001.pdf]

Electronic Supplementary Material (ESI) for RSC Advances.
This journal is © The Royal Society of Chemistry 2023

Supplementary materials for

Effect of cation and anion sizes of additive ionic liquid on the crystal structure of poly(vinylidene fluoride) nanofiber

Hanako Asai*, Hiroyuki Saga, Ryuto Saito, and Koji Nakane

Frontier Fiber Technology and Science Course, Graduate School of Engineering, University of Fukui,

3-9-1 Bunkyo, Fukui, 910-8507, Japan

*Correspondence: Hanako Asai (E-mail: h_asai@u-fukui.ac.jp)

Table S1 Correspondence between mol% and wt% for each IL.

Li^+TFSI^-		EMI^+TFSI^-		BMI^+TFSI^-		$DecMI^+TFSI^-$		EMI^+Cl^-		$EMI^+BF_4^-$	
mol %	wt %	mol %	wt %	mol %	wt %	mol %	wt %	mol %	wt %	mol %	wt %
0.03	0.10	0.05	0.25	0.04	0.25	0.04	0.25	0.03	0.07	0.04	0.10
0.06	0.25	0.09	0.50	0.09	0.50	0.07	0.50	0.09	0.18	0.09	0.25
0.13	0.50	0.19	1.00	0.18	1.00	0.15	1.00	0.19	0.38	0.19	0.50
0.26	1.00	0.28	1.50	0.26	1.50	0.22	1.50	0.28	0.55	0.28	0.75
0.39	1.50	0.38	2.00	0.35	2.00	0.30	2.00	0.38	0.75	0.38	1.01
0.52	2.00	0.46	2.40					0.51	1.01	0.56	1.50

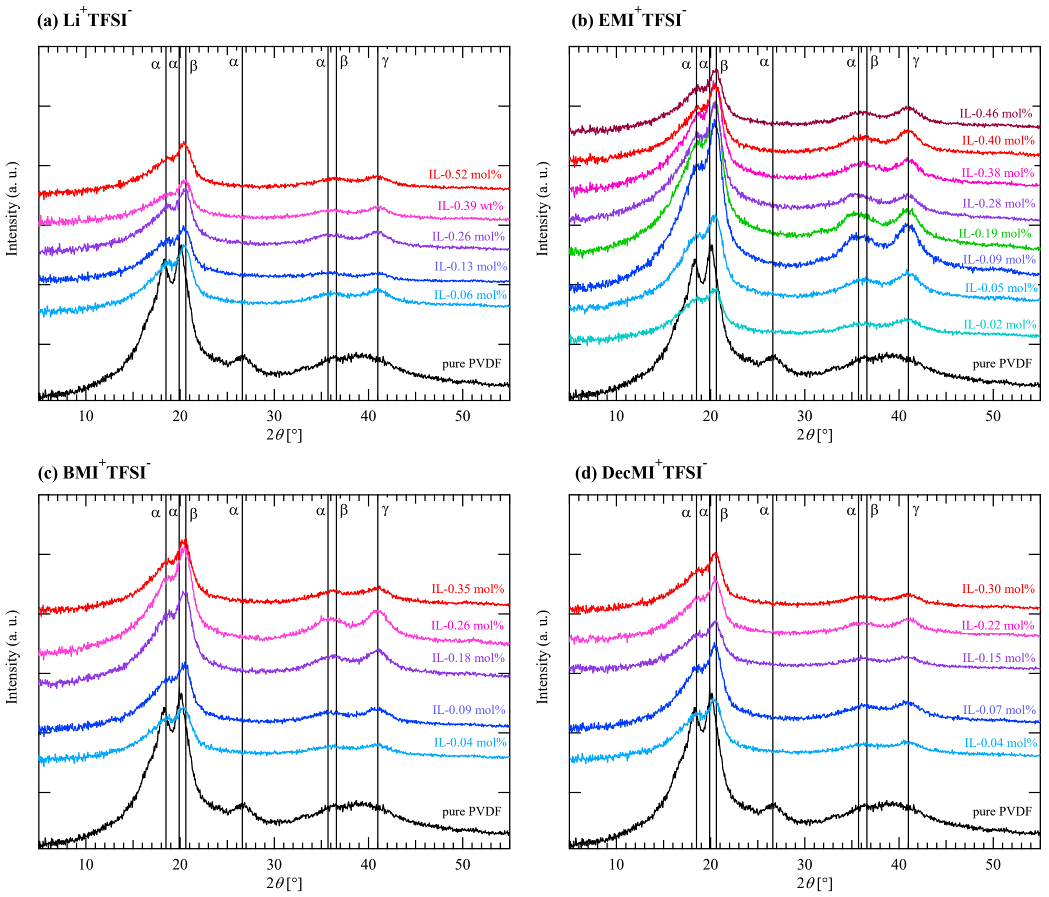

Figure S1 XRD for cation-varied series. The broken and dotted lines indicate the α- and β-phases, respectively. The dotted line at 41.7° indicates the γ-phase.

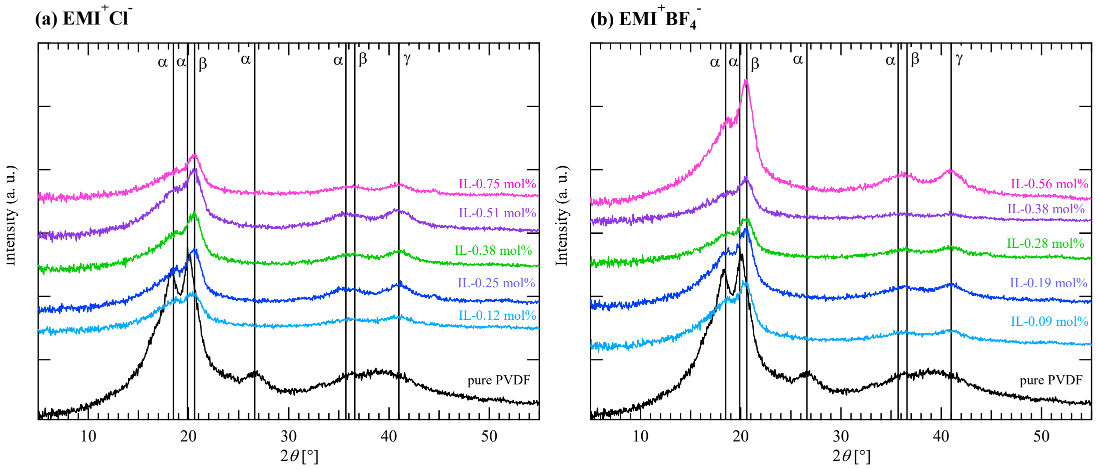

Figure S2 XRD for anion-varied series. The thin and thick broken lines indicate the α- and β-phases, respectively. The dotted line at 41.7° indicates the γ-phase.

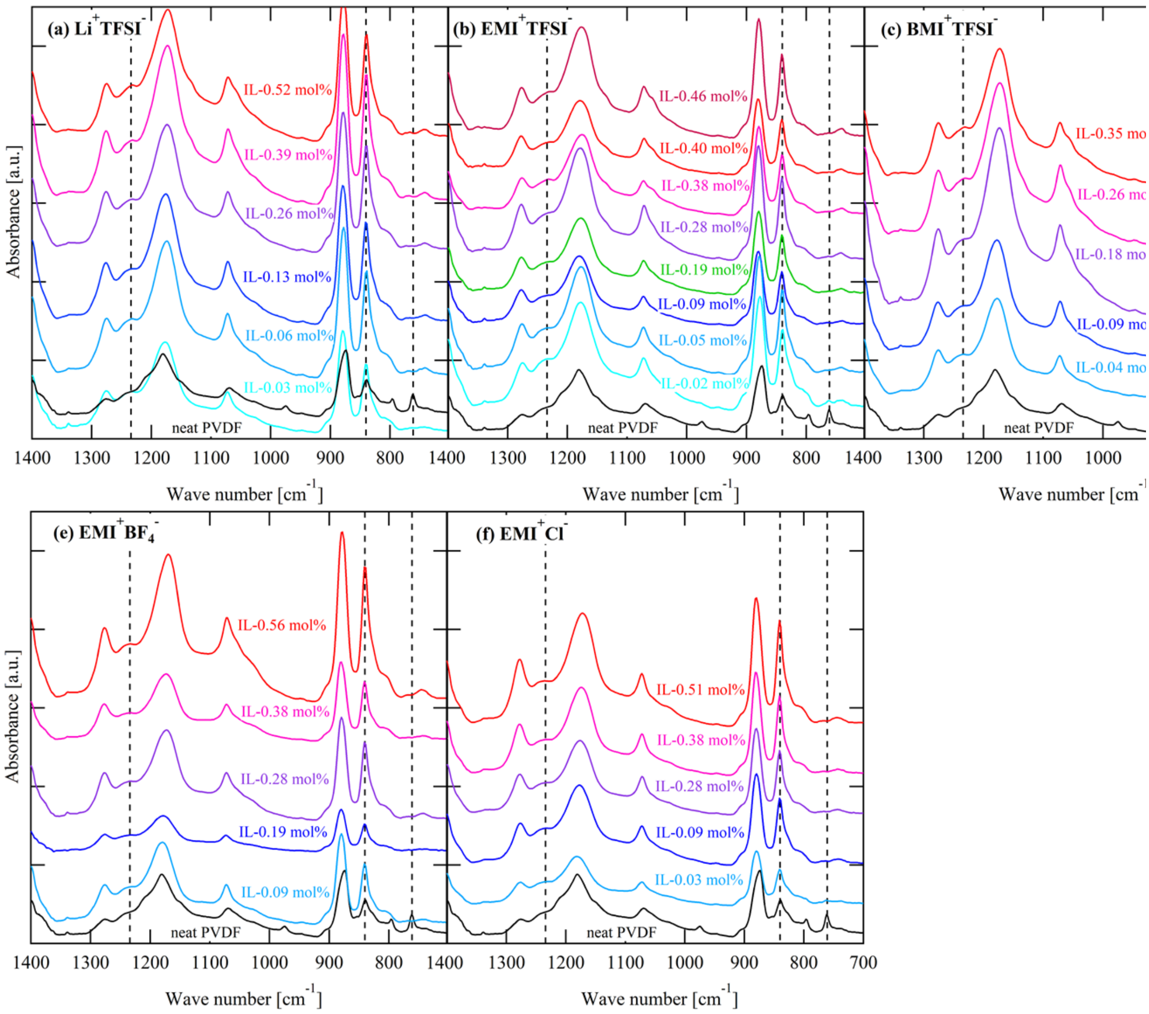

Figure S3 FT-IR spectra for nanofiber samples containing each IL with various concentrations.

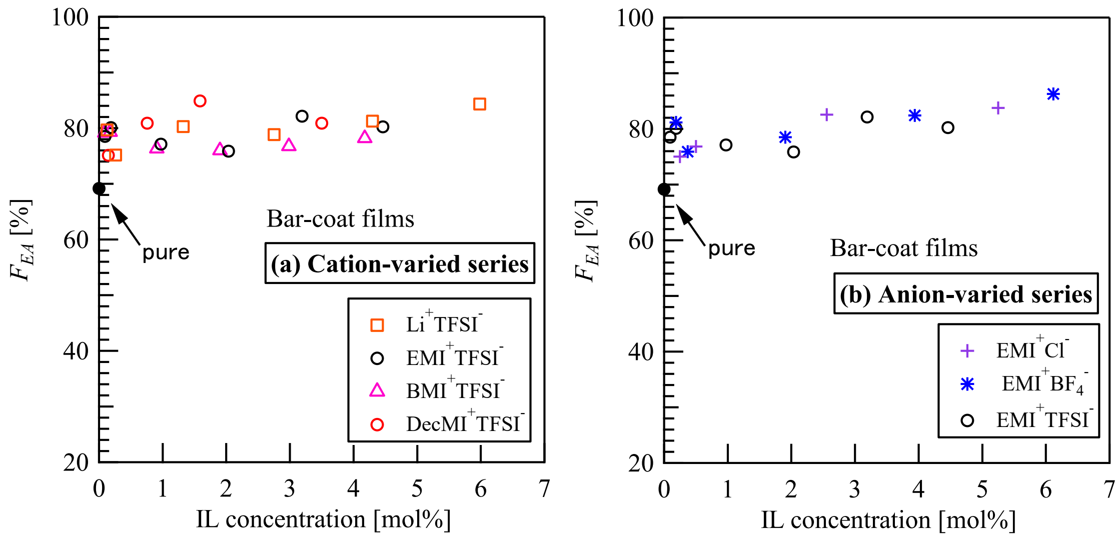

Figure S4 F_{EA} values of the bar-coat film samples evaluated from FT-IR measurements. (a) Cation-varied series, (b) anion-varied series. The film samples were washed by ethanol before the measurements, because the spectrum from the IL around 761 cm^{-1} overlapped with those from the sample (the added IL amount is much larger than the case of nanofiber samples).

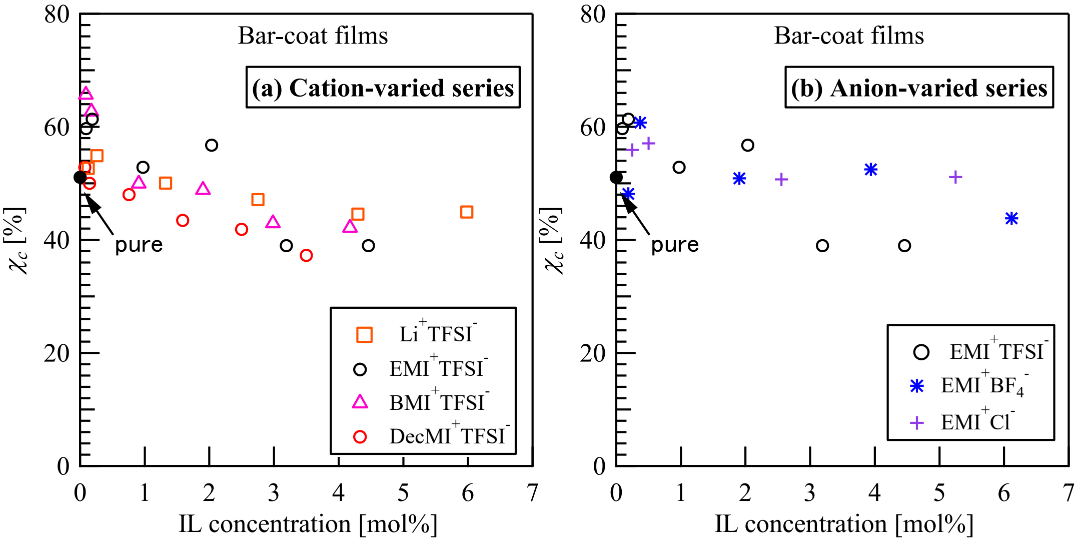

Figure S5 Additive IL concentration dependences on the crystallinity, χ_c of the bar-coat film samples. (a) Cation-varied series and (b) anion-varied series.

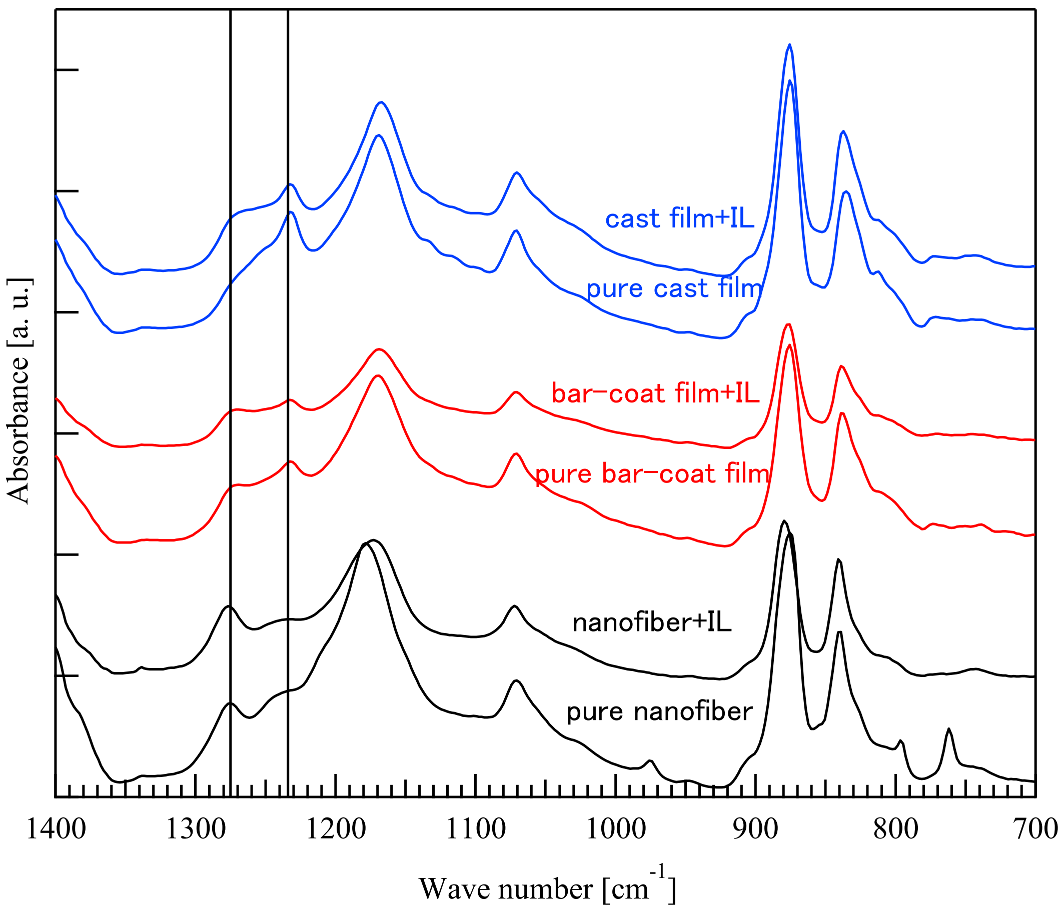

Figure S6 FT-IR spectra for cast films, bar-coat films and nanofibers with and without IL. The added IL was $EMI^+BF_4^-$, and the IL concentration was 0.28 mol%.
